# Malaria: 11‐Year Experience With Imported Cases at a German University Hospital and Epidemiological Trends Amid the COVID‐19 Pandemic

**DOI:** 10.1155/japr/9333826

**Published:** 2026-04-08

**Authors:** Patricia Mester, Claudia Kunst, Stephan Schmid, Katharina Zimmermann, Sigrid Bülow, Janine Rennert, Benedikt M. J. Lampl, Tobias Schilling, Martina Müller, Vlad Pavel

**Affiliations:** ^1^ Department of Internal Medicine I, Gastroenterology, Hepatology, Endocrinology, Rheumatology, and Infectious Diseases, University Hospital Regensburg, Regensburg, Germany, uniklinikum-regensburg.de; ^2^ Department for Clinical Microbiology and Hygiene, University Hospital of Regensburg, Regensburg, Germany, uniklinikum-regensburg.de; ^3^ Department of Radiology, University Hospital Regensburg, Regensburg, Germany, uniklinikum-regensburg.de; ^4^ Regensburg Department of Public Health, Regensburg, Germany; ^5^ Department of Epidemiology and Preventive MedicineFaculty of Medicine, University of Regensburg, Regensburg, Germany, uni-regensburg.de; ^6^ Department of Interdisciplinary Acute, Emergency And Intensive Care Medicine (DIANI), Klinikum Stuttgart, Stuttgart, Germany, klinikum-stuttgart.de

**Keywords:** chemoprophylaxis, COVID-19, imported cases, infectious disease, malaria

## Abstract

**Background:**

By the mid‐20th century, Europe eradicated endemic malaria. However, the significance of imported infections in nonendemic regions is growing. These cases often constitute the majority, can sustain transmission, trigger relapses, and increase mortality in these areas.

**Methods:**

We conducted a retrospective, single‐center study of patients with malaria treated at the University Hospital Regensburg located in the State of Bavaria, Germany, between 2012 and 2023. Our single‐center epidemiological data were analyzed in the context of the COVID‐19 pandemic and compared with overall data from Bavaria, Germany, and Europe.

**Results:**

Fifty‐eight patients with malaria were treated at a German university hospital. The median age at diagnosis was 37.5 years, with 65% of patients being male. A total of 31.6% of the patients required intensive care. This study revealed a survival rate of 98%. Only 17% had taken malaria prophylaxis, and *Plasmodium falciparum* was the causative agent in 96% of cases. The primary laboratory abnormality was thrombocytopenia. Germany reports a significant number of imported malaria cases annually. North Rhine‐Westphalia is the most affected region. The COVID‐19 pandemic impacted global malaria epidemiology, leading to a drastic decrease in malaria cases across Europe, including Germany.

**Conclusion:**

Imported malaria remains rare in Europe. However, severe cases still occur, often requiring intensive care and resulting in high morbidity or even death. The diagnosis and management of malaria in nonendemic settings present a clinical challenge, and the delayed diagnosis is responsible for preventable deaths.

## 1. Introduction

Malaria, a vector‐borne disease, is transmitted through the bite of infected female *Anopheles* mosquitoes. The causative agents, *Plasmodium (P.)* spp., are protozoal parasites within the Plasmodiidae family. In 2021, the World Malaria Report recorded 247 million cases, slightly up from 2020′s 245 million cases. Malaria‐related deaths remained at 619,000 in 2021 compared with 625,000 in 2020. Among over 250 *Plasmodium species*, only four are transmitted by Anopheles mosquitoes from one human host to another: *P. falciparum*, *P. vivax*, *P. ovale* (including subspecies wallikeri and curtisi), and *P. malariae*. *P. falciparum* is a major cause of child mortality worldwide. Additionally, simian parasites such as *P. cynomolgi*, *P. inui*, and especially *P. knowlesi* are responsible for causing human malaria [1–5].

Studies on malaria began in the late 19th century with the emergence of modern microbiology. It became clear that characterizing the disease threatening many parts of Europe was essential. In 1878, French military doctor Alphonse Laveran identified *Plasmodium* gametocytes in patients′ blood using optical microscopy in Bône, Algeria. In 1884, Patrick Manson proposed that mosquitoes acted as vectors for the parasite. In 1899, British army surgeon Ronald Ross confirmed that anopheline mosquitoes transmitted human malaria parasites. Meanwhile, Italian scientists had also demonstrated the transmission of the disease by mosquitoes [1, 5–7].

Malariologists Giovanni Battista Grassi, Amico Bignami, and Giuseppe Bastianelli described the complete cycle of *P. vivax*, *P. falciparum*, and *P. malariae*, recognizing that only *Anopheles* females transmitted the parasites. In 1947, Henry Shortt and Cyril Garnham found preerythrocytic schizonts of *P. cynomolgi* in the liver of a Rhesus monkey, and in 1982, Krotosky explained malaria relapses by deciphering the dormant exoerythrocytic hypnozoites of *P. vivax* [1].

The oldest evidence of *Plasmodium* parasites, dating back 30 million years, was found in amber in the Dominican Republic [8]. Malaria likely coevolved with nonhuman primates worldwide, with *P. falciparum* possibly originating from gorilla parasites around 10,000 years ago and *P. knowlesi* emerging in Southeast Asia among macaque monkeys 478,000–98,000 years ago [9].

Paleomicrobiological investigations in Europe have confirmed *P. falciparum* presence in individuals during the 1st, 2nd, and 5th centuries in Italian regions, later also in Bavaria [10]. The onset of malaria in Europe has various theories. The disease may have been introduced by prehistoric populations moving from India to Europe [4]. Another hypothesis suggests the Carthaginian invasion of Sardinia from North Africa in the 7th to 2nd centuries BC as a possible source [1, 11]. Interestingly, the term “malaria” originates from the Italian word “mal′ aria,” meaning “bad air” [1].

Malaria has historically affected various regions, with Africa bearing the greatest disease burden [12]. During the colonial era, by the 18th century, malaria also caused significant deaths among European colonizers, leading to West and Central Africa earning the epithet “the White Man′s Grave” [13]. Today, Africa remains the most affected continent, with just five countries—Nigeria (24.3%), the Democratic Republic of the Congo (12.51.7%), Uganda (4.7%), Ethiopia (4.7%) and Mozambique (3.6%)—accounting for over half of all global malaria deaths [12] (Figure S1) [14].

In contrast, Europe currently experiences only imported malaria cases (Figure S2) [15]. Malaria reservoirs in Europe have been eliminated since the 20th century. However, prior to World War II, malaria was endemic in several parts of southern Europe, including Portugal, Italy, Greece, and the Balkans [16]. In 1955, the Eighth World Health Assembly, in Mexico, resolved that the World Health Organization (WHO) should initiate and support research and resource coordination to achieve global malaria eradication (Resolution WHA8.30) [17]. Following this decision, WHO launched the Global Malaria Eradication Program, which successfully eliminated malaria from various regions, including Europe [18].

By 1974, the last indigenous cases occurred in Macedonia [19]. In the late 1980s and early 1990s, some regions, including the Caucasus, Central Asian republics, and to a lesser extent, the Russian Federation, saw the reestablishment of local malaria transmission. Turkey also experienced a surge in malaria cases during the 1990s, reporting over 84,000 cases [20].

In response to increasing indigenous cases and malaria outbreaks, the Roll Back Malaria strategy [21] was introduced in affected European countries in 1999, leading to a significant reduction in cases. Subsequently, countries including Armenia, Azerbaijan, Georgia, Kazakhstan, Kyrgyzstan, the Russian Federation, Tajikistan, Turkey, Turkmenistan, and Uzbekistan committed to malaria elimination by 2015 through the Tashkent Declaration in 2005. This declaration resulted in a new regional strategy and in a continuous decline of malaria cases. Although isolated cases and localized outbreaks were reported in Georgia, Greece, and Turkey in 2011 and 2012, in 2015, all European Region countries reported zero indigenous malaria cases, making Europe the first region worldwide to achieve interruption of indigenous malaria transmission [22]. Nevertheless, Europe remains susceptible to ongoing case importation from endemic regions, constituting a constant risk of reestablishment of endemic transmission.

This study is aimed at analyzing the clinical, laboratory, and parasitological characteristics of patients treated for imported malaria at a German university hospital over 11 years. Additionally, it compares the epidemiological trends of malaria in Bavaria, Germany, Europe, and globally before, during, and after the COVID‐19 pandemic.

## 2. Methods

### 2.1. Study Design and Patient Characteristics

This is a university single‐center retrospective study of patients with malaria. All patients were treated between January 2012 and June 2023 at the University Hospital Regensburg in Bavaria, Germany.

### 2.2. Clinical Data and Statistical Analysis

Age, gender, symptoms, disease progression, laboratory and microbiological results, and epidemiological data related to the geographical origin of imported malaria cases were obtained from the medical records system of the university hospital. Statistical analysis (Mann–Whitney *U* test, multivariate regression) was performed using SigmaPlot 15.

### 2.3. Parasitological Diagnosis of Malaria

Laboratory diagnosis was performed using immunochromatographic assay (BinaxNOW Malaria, Abbott, Abbott Park, Illinois, United State), microscopy of Giemsa‐stained thick and thin blood smear examination as well as PCR testing for *Plasmodium* sp. In case of positive detection of *Plasmodium sp.* in either of the three tests, species differentiation was made based on examination of the blood smears as well as additional species‐specific PCRs.

### 2.4. Epidemiological Data

Epidemiological data reported in text or shown in figures were gathered from databases including the World Health Organization, Centers for Disease Control and Prevention, European Centre for Disease Prevention and Control, and the Robert Koch Institute, Berlin, Germany (via SurvStat@RKI 2.0). Patient information was anonymized for analysis, and geographic maps were generated using MapChart.

### 2.5. Literature Review

We performed a systematic search on PubMed, identifying relevant epidemiological and clinical trials regarding malaria during the COVID‐19 pandemic. These studies were then analyzed and compared with our data. Table 1 offers an overview of the literature regarding the incidence of malaria in the context of the COVID‐19 pandemic.

**Table 1:** Literature overview of studies regarding the epidemiology of malaria in the context of the COVID‐19 pandemic.

Study name	Authors, year	Country/city/region
The Impact of COVID‐19 on Implementation of Mass Testing, Treatment and Tracking of Malaria in Rural Communities in Ghana: A Qualitative Study	Cheng et al. 2022 [23]	Ghana
Malaria Diagnosis in an Emergency Department Before and After the COVID‐19 Pandemic: A Retrospective Study	Demotier et al. 2023 [24]	France, Reims
Sustaining Surveillance as an Intervention During the COVID‐19 Pandemic in Cabo Verde and Implications of Malaria Elimination	DePina et al. 2022 [25]	Cabo Verde
Malaria Incidence and Mortality in Zimbabwe During the COVID‐19 Pandemic: Analysis of Routine Surveillance Data	Gavi et al. 2021 [26]	Zimbabwe
Malaria and the Incidence of COVID‐19 in Africa: An Ecological Study	Habibzadeh 2023 [27]	53 African countries
Millions of Excess Cases and thousands of Excess Deaths of Malaria Occurred Globally in 2020 During the COVID‐19 Pandemic	Liu et al. 2022 [28]	Africa, South America, Asia
Implementation and Challenges to Preventing the Re‐Establishment of Malaria in CHINA in the COVID‐19 Era	Maharaj et al. 2023 [29]	China
The Effect of the COVID‐19 Lockdown on Malaria Transmission in South Africa	Prabhu et al. 2022 [30]	South Africa
Malaria Epidemiology and COVID‐19 Pandemic: Are They Interrelated?	Norman et al. 2022 [31]	India
Indirect Effects of the COVID‐19 Pandemic on Malaria Intervention Coverage, Morbidity, and Mortality in Africa: A Geospatial Modelling Analysis	Zhu et al. 2022 [32]	Africa
Trends in Imported Malaria During the COVID‐19 Pandemic, Spain (+Redivi Collaborative Network)	Bylicka‐Szczepanowska et al. 2022 [33]	Spain
An Epidemiological Analysis of Imported Malaria in Shanghai During a COVID‐19 Outbreak	Hakizimana et al. 2022 [34]	Shanghai
Asymptomatic Malaria Infections in the Time of COVID‐19 Pandemic: Experience From the Central African Republic	Namuganga et al. 2021 [35]	Central African Republic
The Impact of Covid‐19 on Malaria Services in Three High Endemic Districts in Rwanda: A Mixed‐Method Study	Reuling et al. 2018 [36]	Rwanda
Impact of COVID‐19 on Routine Malaria Indicators in Rural Uganda: An Interrupted Time Series Analysis	Bhalla et al. 2008 [37]	Uganda

## 3. Results

### 3.1. Study Population

Between January 2012 and June 2023, 58 patients with malaria were treated in our university hospital. The median age of the patients at diagnosis was 37.5 years [18–22, 36–77], and there was a predominance of males (37 patients, 64%). More than half of the study population (32 patients, 55%) had African or Asian roots and acquired malaria while visiting friends and relatives (VFR) in endemic regions. Eighteen patients (31%) had at least one episode of malaria reinfection in the past, and three of them suffered from a second infection during our 11‐year survey. Four patients (7%) in our study cohort suffered from three episodes of malaria. All patients presented to the emergency unit complaining of unspecific symptoms such as fever, headache, and chills. The characteristics of the patients are depicted in Table 2.

**Table 2:** Clinical development of patients with malaria and characteristics of the patient cohort.

Parameter	Patients (*n* = 58)
Age (years): median (range)	37.5 (18–64)

Sex: *n* (%)	
Female	21 (36)
Male	37 (64)
	Clinical development (*n* = 58)

Total days of hospitalization (median, range)	5.5 (1–31)
Days of ICU stay (median, range)	2 (1–31)
Organ failure (number of patients)	4

Comorbidities	
Liver cirrhosis	1
Arterial hypertension	16
Hypertriglyceridemia	13
Diabetes mellitus	8

Survival/death	
Survival (number of patients)	57
Death (number of patients)	1

**Figure 1:**
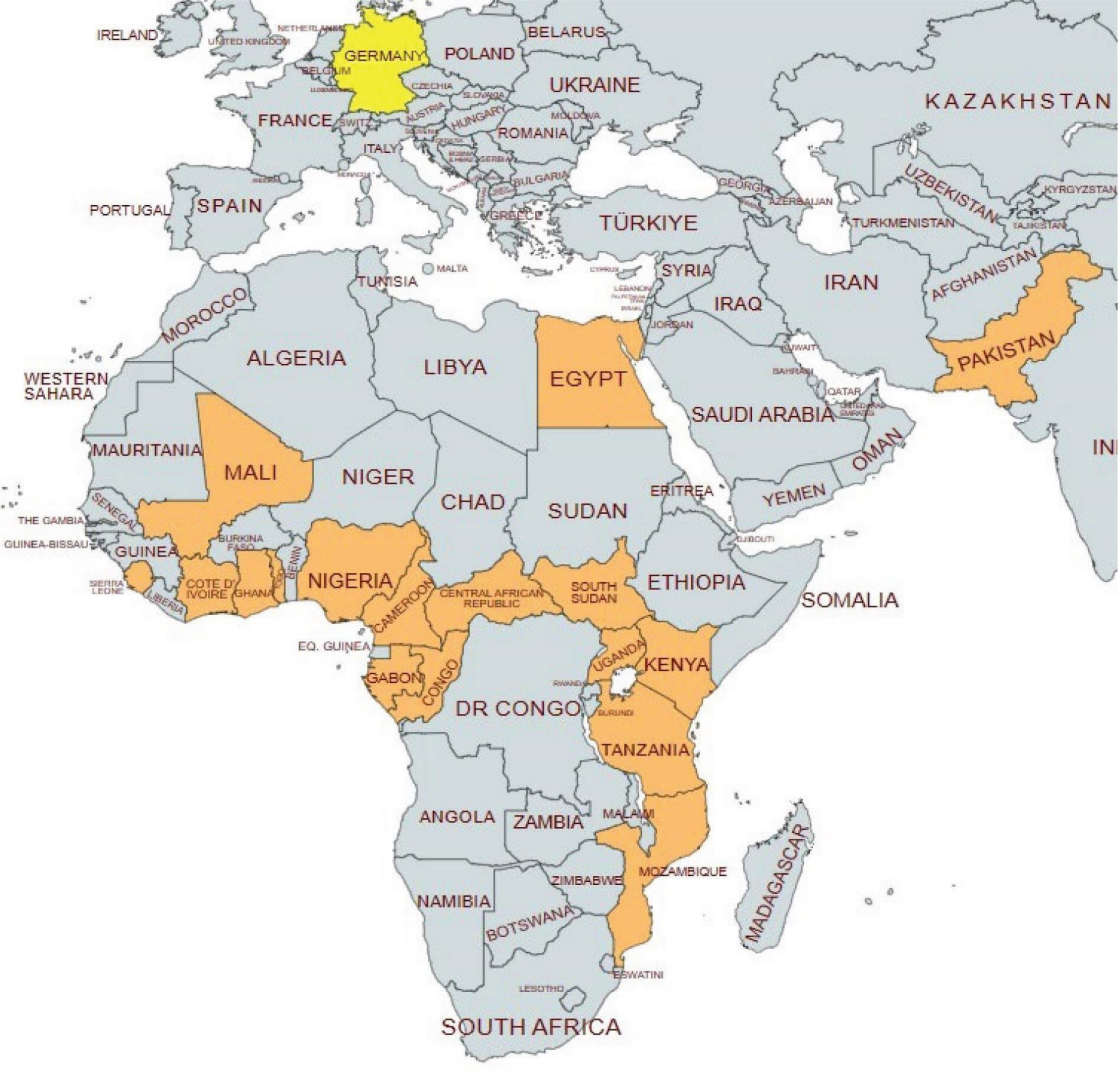
Origins of imported malaria cases (in orange) treated in a German university department from January 2012 until June 2023.

Almost all patients (95%) in our cohort contracted malaria in Africa, with 93% of them infected in the highly endemic Sub‐Saharan region. Specifically, visits to Cameroon and Nigeria accounted for 33% (19 cases) of malaria infections. However, in three patients, the precise infection location remains unknown as they traveled to multiple African countries before symptom onset. Outside of Africa, two patients in our study group contracted malaria in the endemic country of Pakistan. The median number of days spent in these countries was 16 (with a minimum of 9 days and a maximum of 23 days). Furthermore, 38 patients traveled only to urban areas, whereas 20 were exposed also to rural environments. Figure 1 and Table 3 provide representations of the travel destinations of our patients.

**Table 3:** Travel destinations of patients who were subsequently treated for malaria in our clinic.

Country	Number of patients (3 patients traveled in several countries)
Cameroon	10
Nigeria	9
Ghana	6
Kenya	7
Tanzania	5
Togo	3
Congo	3
Uganda	4
Pakistan	2
Mali	2
South Sudan	3
Central African Republic	1
Egypt	2
Gabon	1
Ivory Coast	1
Mozambique	2
Sierra Leone	1
Botswana	1

Out of the 58 patients, 18 (31%) were admitted to the intensive care unit (ICU). The duration of hospitalization varied based on disease severity, ranging from 1 day of observation to 38 days of complex intensive care therapy. The median hospital stay was 5.5 days, and the median duration of ICU treatment was 2 days, with most cases requiring only brief ICU monitoring. However, in two severe cases with multiple organ failure, ICU stays extended over several weeks. Following WHO criteria [36], our study cohort had four patients (7%) classified as experiencing severe malaria, with two (4%) of them needing mechanical ventilation due to cerebral symptoms, reduced consciousness, and respiratory insufficiency (Table 2). Our patient cohort showed a low mortality rate, with 98% (57 patients) surviving, and only one patient (2%) with severe malaria did not survive the disease. Of note, the deceased patient suffered also from liver cirrhosis. Further comorbidities were arterial hypertension (16 cases), hypertriglyceridemia (13 cases), and diabetes mellitus (8 cases) (Table 2).

### 3.2. Microbiology of Malaria Species in our Cohort

In our study group, 46 patients (79%) contracted *P. falciparum* (Figure 2a,b), whereas *P. malariae* (Figure 2c), *P. ovale* (Figure 2d), and *P. vivax* (Figure 2e) were responsible for infections in four (7%), three (5%), and three (5%) patients, respectively. Additionally, two patients (4%) had mixed infections (Table 4). Most patients with falciparum malaria had an initial parasitemia below 5%, only in four cases a parasitemia above 5% could be detected. Median parasitemia levels of severe cases was 12.5% (2%–30%). Of clinical relevance, only 10 patients (17%) took prophylactic measures. The prophylactic medication used included atovaquone/proguanil (three cases), mefloquine (three cases), artemether/lumefantrine (two cases), chloroquine (one case), and doxycycline (one case).

Figure 2(a and b) *P. falciparum* trophozoits in the thin blood smear (a) and *P. falciparum* gametocytes in thick blood smear (Giemsa stain), (b) parasitemia 2.0%, immunochromatography positive, PCR positive for *P. falciparum*. *P. malariae*, Giemsa, thin blood smear. (c) Only isolated parasites found, immunochromatography negative, PCR positive for *P. malariae*. (d) *P. ovale* in thin blood smear (Giemsa stain), parasitemia 0.5%, immunochromatography negative, PCR positive for *P. ovale*. (e) *P. vivax* in thin blood smear (Giemsa stain), parasitemia 0.2%, immunochromatography positive, PCR positive for *P. vivax.*

**Figure:**
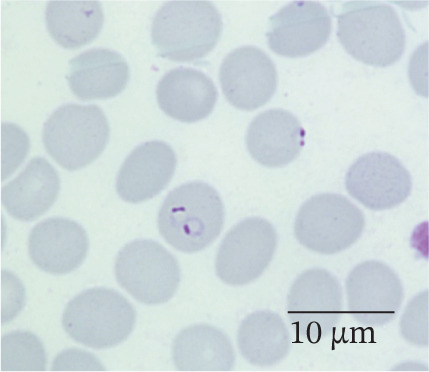
(a)

**Figure:**
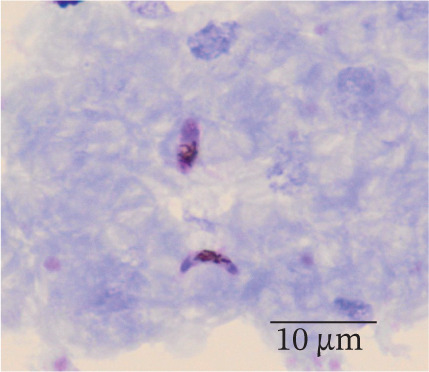
(b)

**Figure:**
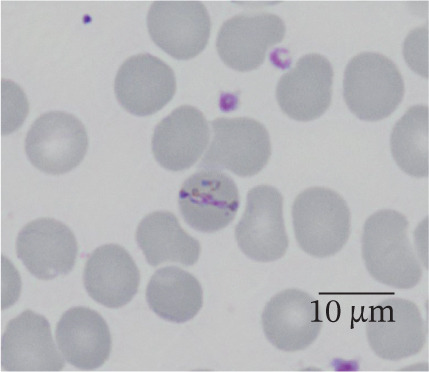
(c)

**Figure:**
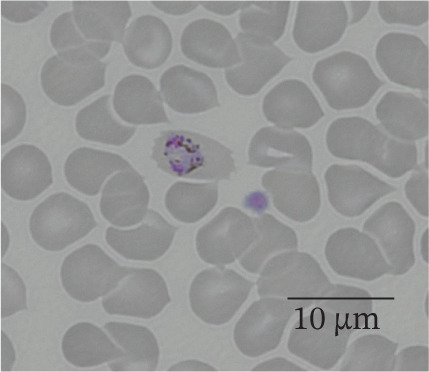
(d)

**Figure:**
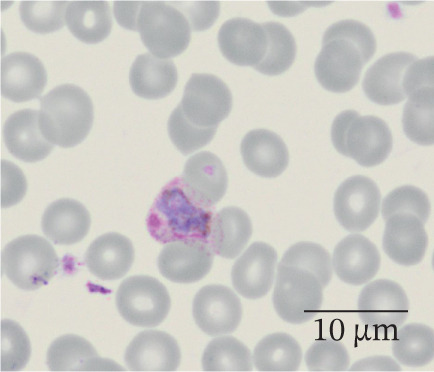
(e)

**Table 4:** Microbiology of malaria species, recurrence rates and prophylaxis in our cohort.

Plasmodium type	Patients *n* (%)
*P. falciparum*	46 (79)
*P. malariae*	4 (7)
*P. vivax*	3 (5)
*P. ovale*	3 (5)
Mixed infection	2 (4)

Recurrence:	**Patients n (%)**
One	15 (25)
Two	3 (5)

Parasitemia levels of severe cases	**12.5% (2%–30%)**
Prophylaxis	**Patients n (%)**
Yes	10 (17)
No	48 (83)
Mefloquine	3 (5)
Atovaquone/proguanil	3 (5)
Artemether/lumefantrine	2 (4)
Chloroquine	1 (2)
Doxycyclin	1 (2)

### 3.3. Initial Clinical Presentation in Malaria

Most of our patients presented to the emergency unit with nonspecific symptoms, with most experiencing fever as the primary complaint. In four cases with severe malaria, patients presented with impaired consciousness and dyspnea.

### 3.4. Laboratory Changes in Malaria

**Figure 3:**
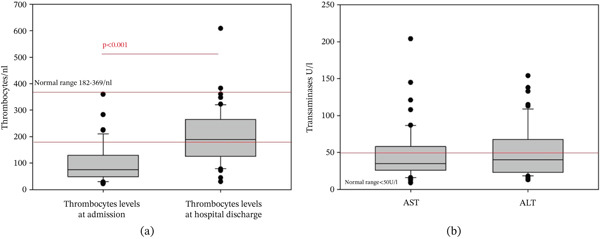
(a) Levels of thrombocytes and transaminases in patients with malaria. Thrombocytopenia was the main pathological laboratory finding in our cohort. Thrombocytes levels improved significantly after specific antimalaria treatment. (b) At admission, AST and ALT levels were increased in 26% and 35% of cases, respectively.

In our cohort, thrombocytopenia represented the primary abnormality of the laboratory results, affecting 46 patients (79%), with 89% of them infected with *P. falciparum* (Figure 3a). Of note, with appropriate antimalarial treatment in accordance with current guidelines, platelet levels showed significant improvement, as illustrated in Figure 3a.

Additionally, elevated aspartate transaminase (AST) was observed in 15 patients (26%), whereas alanine transaminase (ALT) elevation occurred in 20 patients (35%) (Figure 3b), although it tends to be overlooked in uncomplicated cases [36]. Although hepatic involvement in uncomplicated malaria cases is relatively rare [37], our findings suggest that monitoring liver enzyme levels is advisable for patients with suspected or confirmed malaria.

**Figure 4:**
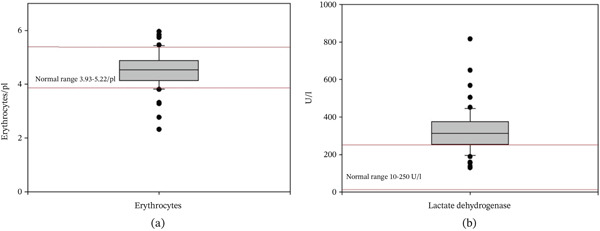
Levels of (a) erythrocytes and (b) lactate dehydrogenase (LDH) in patients with malaria. Anemia was present in 16% of cases at admission. However, 79% of cases had increased LDH levels, suggesting hemolysis.

Twelve patients (21%) exhibited elevated creatinine levels (data not shown), whereas nine patients (16%) presented with anemia (Figure 4a). Of clinical importance, in 45 cases (78%), lactate dehydrogenase (LDH) levels were elevated, indicative of hemolysis (Figure 4b). Multivariate regression analysis identified that increased levels of bilirubin and AST correlated to decreased levels of thrombocytes and hemoglobin and elevated INR value at admission as predictive factors for severe disease (*p* = 0.001). A further comparative analysis between prepandemic and pandemic cases did not find any statistical differences (thrombocytes *p* = 0.220, bilirubin *p* = 0.508, AST *p* = 0.324, ALT *p* = 0.421, creatinine *p* = 0.421, hemoglobin *p* = 0.844).

### 3.5. Trends in the Epidemiology of Malaria and the Influence of the COVID‐19 Pandemic in Europe

Throughout the 20th century, Europe successfully eradicated its malaria reservoirs [16]. Nevertheless, the continent continues to face malaria, primarily through imported cases (Figure 5a) [38]. Notably, in 2021, 99.7% of these cases were attributed to travel‐related transmission (Figure S2) [15]. We analyzed epidemiological data from databases including the World Health Organization, Centers for Disease Control and Prevention, European Centre for Disease Prevention and Control, and the Robert Koch Institute. These data show that France consistently records the highest number of imported malaria cases in Europe, with approximately 5500 cases reported annually, as stated by the Pasteur Institute [14]. Additionally, countries such as Germany, Italy, Spain, and Belgium also report significant numbers of imported malaria cases each year. In 2021, Germany ranked as the second‐highest country in the European Union for imported malaria cases, with 605 reported cases (Figure 5b) [15]. From August 2021 to August 2022, the state of North Rhine‐Westphalia in Germany saw the highest impact, reporting 259 cases of imported malaria. Following closely, Baden‐Wuerttemberg reported 127 cases and Berlin 104 cases. Bavaria recorded 95 cases during the same period (Figure S3). Within Bavaria, the district of Upper Bavaria was the most affected, with 49 cases. The district of Upper Palatinate, where our university center is situated, reported nine cases during the same timeframe (Figure S4). These nine patients were referred to our institution, where from 2021 to 2022, 14 cases were admitted.

Figure 5(a) Thousands of imported malaria cases are reported yearly within the European Union. During the COVID‐19 pandemic, a significantly decreased number of imported malaria cases was observed. (b) Countries most affected by imported malaria in the European Union in 2021.

**Figure:**
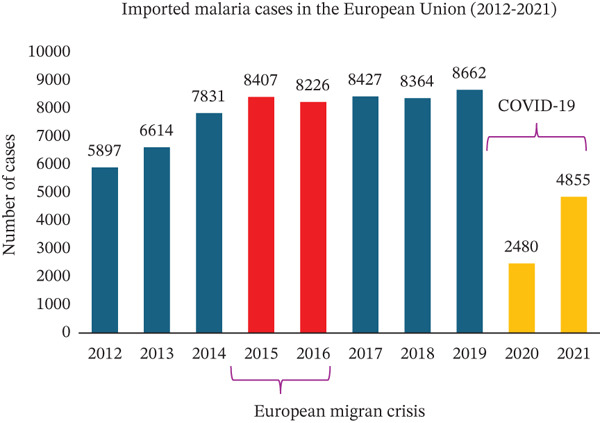
(a)

**Figure:**
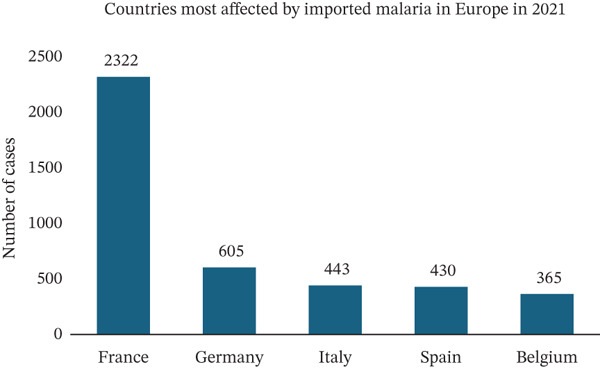
(b)

In the past 11 years, 2015 stood out as the year with the highest number of malaria cases treated in our university department (Figure 6a). This aligns with both European and German national statistics. Starting in 2014, reported malaria cases increased significantly in the European Union and Germany (Figures 5a, 6b) [39], with 2015 marking the highest number of reported cases in Germany in the last 11 years (Figure 6b). This rise in malaria cases can be attributed to the European migrant crisis in 2015 (Figures 5a, 6b) [40]. During 2015 and 2016, Germany saw over 1 million asylum applications [41]. Reported malaria cases remained relatively stable until 2020 when a notable decline was observed [42], possibly due to travel restrictions imposed during the COVID‐19 pandemic. This decreasing trend was observed not only at the European level but also at the national and local levels in Germany (Figure 5a, 6b, 7a, and 7b). Of note, the World Tourism Organization reported a reduction of travel volume up to 72% during the COVID‐19 pandemic [43].

Figure 6Malaria epidemiology (a) in Germany and (b) in Bavaria from 2012 until 2022. During the COVID‐19 pandemic, fewer cases were documented.

**Figure:**
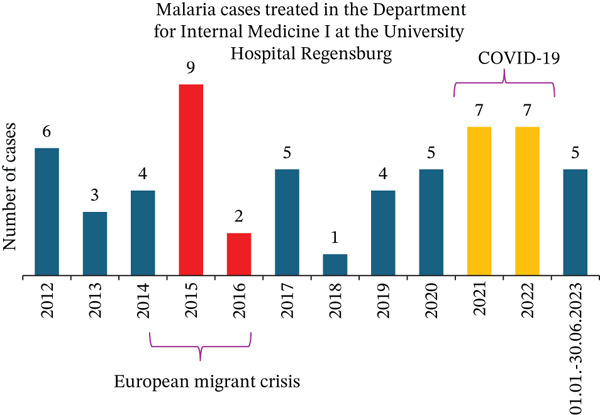
(a)

**Figure:**
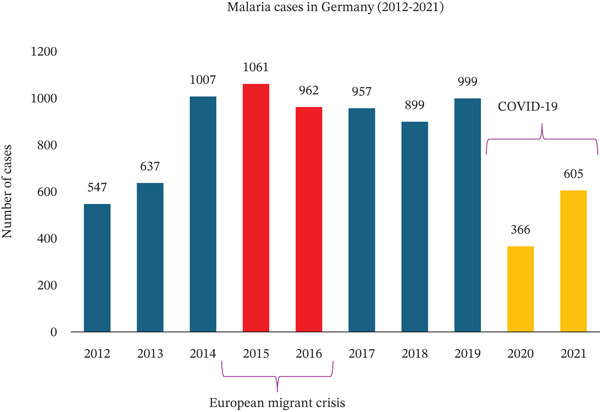
(b)

Figure 7(a) Malaria epidemiology in the Bavarian district of Upper Palatinate from 2012 until 2022. (b) Malaria cases treated at the University Hospital Regensburg from January 2012 until Juin 2023.

**Figure:**
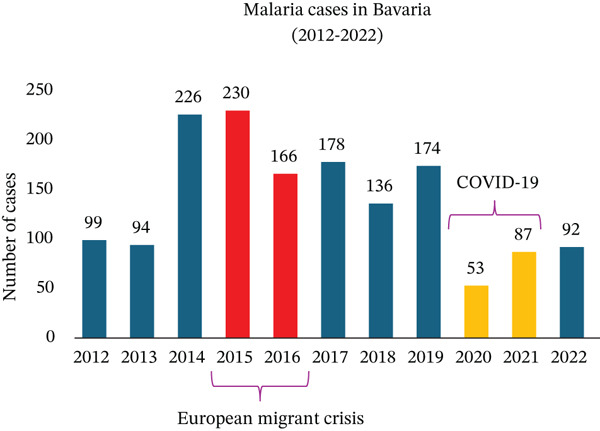
(a)

**Figure:**
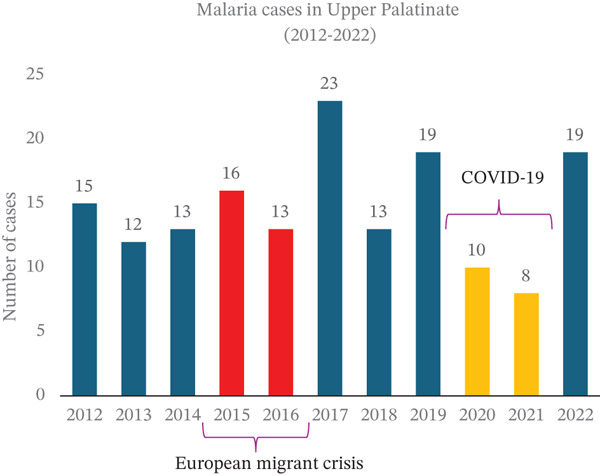
(b)

Since 2012, Bavaria has reported 1535 cases of malaria, with the majority being male patients (1129, 74%) (Figure 8a). In terms of age distribution, 1285 patients (84%) fell in the 15–59‐year‐old range. Children accounted for 127 cases (8%), whereas elderly individuals represented 123 cases (8%) (Figure 8b).

Figure 8(a) Gender and (b) age distribution in the patient cohort of the Department of Internal Medicine I at the University Hospital Regensburg.

**Figure:**
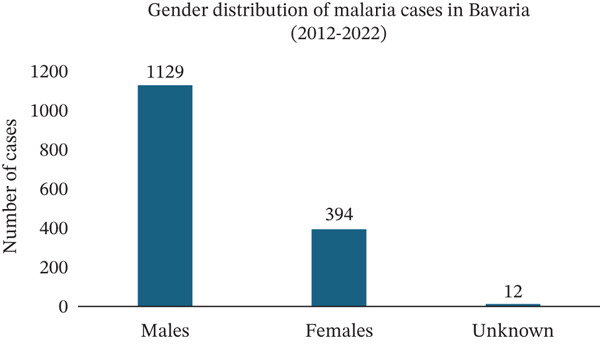
(a)

**Figure:**
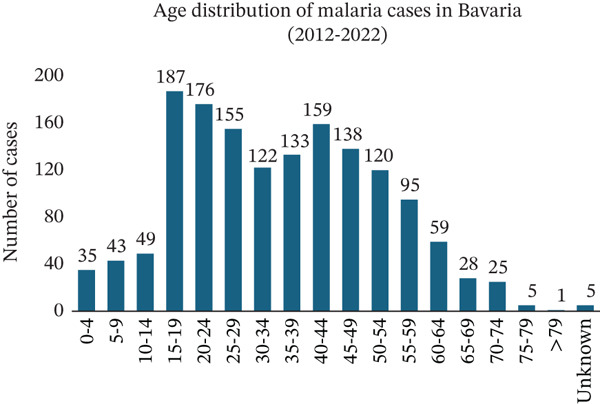
(b)

**Figure 9:**
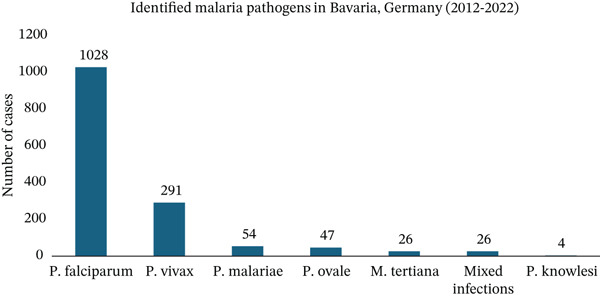
Identified malaria species in Bavaria from 2012 until 2022.

Among the cases reported in Bavaria since 2012, 1028 (67%) were attributed to *P. falciparum*, followed by 291 cases (19%) of *P. vivax* infections. Mixed infections were identified in only 26 cases (1.7%) (Figure 9).

Of note, our department handled a higher number of malaria cases during the COVID‐19 pandemic than in the years before the pandemic (Figure 6a). As the COVID‐19 pandemic caused serious strains and rearrangements to health care systems and infrastructure [44], we hypothesize that this increase could be linked to the reorganization of the healthcare system during the pandemic, where certain hospitals primarily focused on treating COVID‐19 cases, resulting in a greater referral of patients with other conditions to tertiary centers. To address this issue, additional organizational studies should be performed.

## 4. Discussion

The present study describes clinical, epidemiological, and parasitological characteristics of 58 patients with imported malaria treated at a German university hospital over a time span of 11 years. In our cohort, 65% of patients were male, and in 96% of cases *P. falciparum* was identified. Thirty‐three percent of the cases acquired the disease in Cameroon and Nigeria. Seven percent of patients fulfilled the WHO criteria for severe malaria. Most of the patients presented with mild symptoms, but in severe cases impaired consciousness and dyspnea were observed. Noteworthy, in one patient cerebral edema and cytotoxic lesions of the corpus callosum (CLOCC) were detected in cephalic imaging. However, the lesions disappeared with the improvement of the patient′s condition (Figures S5 and S6). To our knowledge, only two case reports described CLOCC syndrome in malaria so far [45, 46]. CLOCC syndrome has been associated also with other infectious diseases, metabolic disturbances, malignancies, or traumatic brain injuries [47]. The pathophysiology behind this syndrome remains uncertain. Intramyelinic edema, oxidative stress, or neuroaxonal damage are possible explanations [47]. Thrombocytopenia was present in 79% of cases; however, under adequate antimalaria treatment, platelet levels improved significantly. Of clinical relevance, our results show that liver enzymes are elevated in about one‐third of cases, suggesting liver damage. Thus, liver biomarkers should be tested even in less severe cases of malaria.

We observed poor adherence regarding chemoprophylaxis, with only 18% of patients taking antimalaria medication before traveling to endemic areas. One patient died (2%), possibly due to malaria complications. An overview of the mortality rates reported by other authors is given in Tables 5, 6.

**Table 5:** Mortality rates in unicentric studies.

Study (unicentric)	Period	Number of total cases	Number of severe cases	Number of deceased patients	Country
Bruneel et al.	1988–1999	188	93	10 (5.3%)	France
Marks et al.	1994–2010	1616	124	5 (0.3%)	United Kingdom
Elawad et a*l.*	1990–1996	112	Nd	0 (0.0%)	United Kingdom
Nathwani et al.	1980–1991	110	Nd	0 (0.0%)	United Kingdom
Gonzalez et *al*.	1991–2007	20	20	5 (25.0%)	Spain
Lopez‐Velez et al.	1989–1995	160	17	0 (0.0%)	Spain
Santos et al.	1990–2011	284	59	9 (3.2%)	Portugal
Ruas et al.	2006–2016	225	1	Nd	Portugal
Antinori et al.	1998–2007	291	35	0 (0.0%)	Italy
Antinori et al.	2010–2015	180	9	0 (0.0%)	Italy
Calderaro et al.	2013–2017	87	0	0 (0.0%)	Italy
Losert et al.	1992–1999	69	7	3 (4.4%)	Austria
Strauss et al.	1990–2000	924	Nd	12 (1.3%)	Austria
Schwake et al.	1996–2003	165	34	Nd	Germany
Vygen‐Bonnet et al.	2001–2016	10256	Nd	51 (0.5%)	Germany
Roggelin et al.	2011–2013	200	Nd	Nd	Germany
Demotier et al.	2011–2021	306	Nd	Nd	France
Yayan et al.	2004–2012	15	Nd	0 (0.0%)	Germany

Abbreviation: Nd, not described.

**Table 6:** Mortality rates in multicentric studies.

Study (multicentric)	Period	Number of total cases	Number of severe cases	Number of deceased patients	Country
Bruneel et al.	2000–2006	400	400	42 (10.5%)	France
Badiaga et al.	1996–2002	42	5	3 (7.1%)	France
Checkley et al.	1987–2006	39302	Nd	191 (0.5%)	United Kingdom
Smith et al.	1987–2006	39300	Nd	157 (0.4%)	United Kingdom
Norman et al.	2009–2016	850	Nd	0 (0.0%)	Spain
Sabatinelli et al.	1989–2002	1941	Nd	26 (1.3%)	Italy
Thierfelder et al.	1994–2004	109	14	0 (0.0%)	Switzerland
Giannone et al.	1990–2019	8,439	Nd	52 (0.6%)	Switzerland
Normal et al.	2009–2021	1751	64	4 (0.2%)	Spain
Gavi et al.	2017–2020	909,274	Nd	2029 (0.2%)	Zimbabwe
Hakizimana et al.	2020	Nd	Nd	No exact number reported	Rwanda
Bylicka‐Szczepanowska et al.	2021	515	Nd	Nd	Central African Republic
Maharaj et al.	2015–2020	Nd	Nd	Nd	South Africa
Namuganga et al.	2017–2021	1,442,737	Nd	Nd	Uganda
Cheng et al.	2022	Nd	Nd	Nd	Ghana
DePina et al.	2017–2020	31,040	Nd	Ndd	Cabo Verde
Habibzadeh et al.		Nd	Nd	Nd	Africa
Prabhu et al.	2000–2020	Nd	Nd	Nd	India
Zhu et al.	2020–2021	112	15	0 (0.0%)	China
Lu et al.	2020–2021	1	Nd	Nd	China
Torres et al.	2018–2020	7772	Nd	1769 (22.8%)	Peru
Liu et al.	2000–2020	Nd	Nd	Nd	Africa
Kendjo et al.	1996–2016	43,333	2462	163 (0.4%)	France
Kurth et al.	2006–2014	185	185	3 (1.6%)	12 European countries

Abbreviation: Nd, not described.

Epidemiological data revealed that during the COVID‐19 pandemic fewer malaria cases were documented in Germany and in Europe overall. However, according to the Robert Koch Institute, a significant increase in fatal cases linked to malaria was described in 2022 in Germany, when nine deaths were reported. All these patients were infected with *P. falciparum*. Since 2006, a maximum of four deaths per year have been reported. Paradoxically, our department treated more malaria cases in this period. Twenty‐two cases (38%) were admitted during the pandemic years, one of which was severe. However, few studies regarding imported malaria in Europe during the COVID‐19 pandemic were performed. As already mentioned, this fact could be caused by the reorganization of the healthcare system during the pandemic. To our knowledge the actual study is the third to describe the trend of malaria cases during the pandemic in Europe and is the only European study describing the epidemiology of malaria both at local and national levels, specifically in Bavaria and Germany. Furthermore, it is the only study presenting epidemiologic data of malaria from a tertiary center spanning the entire COVID‐19 pandemic.

Fifty years after its eradication on the continent, malaria remains a major health concern in Europe. Since the 1970th, malaria cases in Europe are commonly travel related and up to 10% of them progress to severe forms [48, 49]. Although sporadic, autochthonous malaria infections still appear in European countries [50–53]. Furthermore, airport malaria cases have been reported by some countries like Belgium and Germany [54, 55]. Moreover, a study from Belgium reported that most travelers presenting with fever are diagnosed with malaria, with falciparum malaria being the only tropical cause of death in the study population [56].

## 5. Comparison of Own Results With European Studies

The most affected country in Europe remains France [3, 14] where over time, a couple of studies concerning patients with imported malaria were performed [49, 57, 58]. Bruneel et al. performed a retrospective study of 188 patients with severe falciparum malaria investigating the clinical spectrum of the disease [57]. Just like in our study, most patients acquired malaria in Sub‐Saharan Africa [57]. Similar to our results, all the patients with less severe malaria survived. However, the mortality in the severe malaria group was 11% (10 patients) and 43% (40 patients) needed mechanical ventilation [57]. Moreover, 31% (29 patients) required dialysis [57]. Furthermore, in the study by Bruneel et al., cerebral imaging revealed abnormalities in 10 patients [57].

Another study by Bruneel et al. including 400 patients with severe falciparum malaria from 45 French ICUs revealed that 96% of patients also acquired the disease in Sub‐Saharan Africa. Intensive care mortality was 10.5% [58]. In this multicentric French study, 23 patients presented cerebral abnormalities in neuroimaging [58]. A total of 33% (132 patients) of cases required mechanical ventilation and 20% of them (81 cases) needed dialysis [58].

The study by Badiaga et al. analyzed the clinical presentation of patients with severe malaria at the time of admission over a 6‐year period. Severe manifestations like ARDS, coma, shock, acidosis, or acute renal failure were present in 12 cases, three of whom died [49]. Noteworthy, 62% of patients presented with jaundice, suggesting hepatic involvement [49]. Furthermore, both studies by Bruneel et al. showed elevated bilirubin levels in patients with severe falciparum malaria upon admission [57, 58]. Furthermore, Antinori et al. also report elevated transaminases in malaria patients [59, 60]. Although most of the cases in our cohort suffered from less severe forms of malaria, in about a third of cases laboratory findings revealed elevated transaminases suggesting hepatic injury. Moreover, one severe case in our cohort developed liver failure with nonmeasurable coagulation parameters requiring substitution. These findings highlight the importance of measuring liver biomarkers in patients with suspected or proven malaria. Regarding antimalaria chemoprophylaxis, in line with our findings, the percentage of patients who took chemoprophylaxis was low [49, 57, 58].

Other European studies describe patients with malaria mostly acquired in Sub‐Saharan Africa [59, 61–63]. In accordance with our work, *P. falciparum* is the main pathogen causing malaria in Europe [64–68], and predominantly males are affected by the disease [39, 69].

Besides patients on invasive ventilation or dialysis, studies from Portugal and Austria described malaria cases requiring venovenous extracorporeal membrane oxygenation (ECMO) and inhaled nitric oxide (NO) [67, 70]. Cerebral manifestation of the disease is also described by other European studies; however, CLOCC syndrome has not been reported [70, 71].

Kurth et al. report a median time of hospitalization of 7 days, which is comparable with the median time of hospitalization in our cohort (6 days). Moreover, the median length of stay on the ICU was 3 days, which closely aligns with our findings (2 days) [48].

In accordance with our results, European authors describe thrombocytopenia as the main laboratory disturbance [59, 60, 64]. Furthermore, depending on the number of severe cases included in the studies, mortality rates vary from 0% to 25% [66, 72]. Moreover, Losert et al. report an overall mortality of 4% and an ICU mortality of 40% [67]. We observed an overall mortality of 1.72% in our cohort. However, it is to mention that we had only four severe (WHO) cases in our cohort.

In line with our findings, a poor adherence to antimalaria chemoprophylaxis was observed Europe‐wide [49, 58, 61, 70]. Only a Scottish study observed a higher adherence (70%) to chemoprophylaxis [66]. Reasons for this low prophylaxis rate were already identified as low risk misperception of travelers to acquire the disease, mistrust of advice from healthcare providers, fear of side effects, forgetfulness, travel duration, incorrect information, or inappropriate health advice [73, 74]. Also, political involvement and nonscientifically proven statements probably had relevant implications regarding people′s perceptions of some drugs and their possible side effects [75–77], leading to the low adherence of the population to antimalaria chemoprophylaxis observed during the COVID‐19 pandemic.

A possible explanation of the various statistical results among studies could be the larger patient cohorts that include also an increased number of severe cases. Exemplarily, as France has the highest number of malaria cases in Europe, French studies include larger multicentric cohorts with severe cases, explaining also the higher number of patients with organ failure and the higher mortality rates. Furthermore, one study reports only 20 severe cases but a mortality rate of 25% [72]. Tables 5, 6 highlight the mortality rates in several studies already described in the literature.

Taken all together, imported malaria remains a threat to European countries, and due to possible severe complications like organ failure, lethal outcome, and cerebral manifestation of the disease, patients should be closely monitored in experienced centers.

## 6. Malaria During the COVID‐19 Pandemic

To our knowledge, only two European studies were performed concerning the epidemiology of malaria during the COVID‐19 pandemic. Demotier et al. performed a study regarding the diagnosis of malaria from 2011 until 2021 in France in an emergency department [24]. Similar to our results, the authors report a male predominance in their cohort. Furthermore, the authors report a decrease in the number of thick and thin blood smear examinations performed after the onset of the COVID‐19 pandemic in 2020 and a relative rise in 2021 [24].

A 12‐year study from Spain including data from 22 health centers reported 1751 cases of malaria, also with male predominance [31]. Furthermore, the authors identified *P. falciparum* in 81.3% of the cases. In this study cohort, Norman et al. report 64 severe cases and four fatal cases, all in the prepandemic years [31]. Similar to the French study by Demotier et al., the Spanish authors observed a decrease of malaria cases in 2020. Of note, the present work, in contrast with the studies mentioned above, illustrates detailed epidemiological data at both local–regional and national levels. To the authors′ knowledge, it represents the only European study that analyzes this issue in detail.

These two studies are in accordance with both our local data from Bavaria and national data from Germany. Since the World Tourism Organization reported a significant reduction of travel volume during the pandemic [43], the decrease in malaria cases was probably induced by the implementation of the lockdown and by the reduction in the number of flights to tropical areas. In contrast to these trends, our department treated more malaria patients during the pandemic years 2020, 2021, and 2022 than before (Figure 7b). A possible explanation could be the rushed return of German nationals from endemic areas due to concerns about unforeseeable travel restrictions and, as previously mentioned, the restructuring of the healthcare system during the pandemic.

In contrast to Europe, some countries in Africa experienced an increase in malaria cases during the COVID‐19 pandemic [26, 33, 34], whereas other countries experienced a decrease of cases [29, 35]. According to Gavi et al., a possible explanation for the increase in some countries could be the COVID‐19–related disruptions in the malaria prevention and control activities [26]. Two studies from Ghana and the Central African Republic reinforced this idea [23, 33]. On the other hand, authors from Uganda and Cabo Verde reported that the COVID‐19 pandemic did not have any negative effects on malaria disease prevention and treatment [25, 35].

Another interesting study from Africa revealed that countries with higher prevalence of malaria had lower incidence of COVID‐19, suggesting that malaria could protect people against SARS‐CoV‐2 through the stimulation of innate immunity [27]. This observation was also made in India [30].

Studies from China report a decrease in imported malaria cases since the beginning of the COVID‐19 pandemic [32, 78]. Probably due to the lockdown implemented during the pandemic, China experienced the lowest number of malaria infections in history [32]. On the other hand, the global pandemic situation led to an increased number of individuals returning from overseas. According to Zhu et al., this led to a rapid increase in imported malaria cases [32]. This analysis found that in 2021 Shanghai experienced the highest levels of malaria cases in recent years [32].

Data from South America regarding the incidence of malaria during the COVID‐19 pandemic are scarce. To our knowledge, the only study comes from Peru [79]. In line with the other studies performed in Europe, Asia, and Africa, Peru also experienced a decrease in malaria cases during the pandemic. Even in regions with known high transmission characteristics like the Peruvian Amazon, the cases of malaria decreased remarkably [79]. Moreover, not only did the number of malaria infections decrease but also the number of dengue or leptospirosis cases. According to Torres et al., these findings could be partly due to underdiagnosis [79]. Limitation to public transportation or closing some health care centers during the pandemic could provide an explanation for the decreased numbers of diagnosed malaria cases [79].

Based on data from the World Health Organization Global Observatory, Liu et al. showed that African countries suffered the most during 2020, Nigeria reporting the largest number of malaria cases and deaths [28]. Comparable with our results, the authors report that most countries witnessed a lower number of malaria cases during the COVID‐19 pandemic, with the strongest decline of incidence rates in Bhutan and mortality rates in Ecuador [28].

This study has limitations. First, it highlights the experience with malaria cases from a single center. Furthermore, the patient cohort described is small, especially as the number of severe cases is reduced, and this limits statistical power. Moreover, its descriptive character does not allow clarification of causal issues.

## 7. Conclusion

Despite global progress in combating malaria, malaria continues to cause millions of infections and thousands of deaths annually, with over 90% of fatalities occurring in sub‐Saharan Africa. Although Europe was declared malaria‐free in 1975, imported cases still occur. During the COVID‐19 pandemic, most countries reported fewer malaria cases, but some African endemic regions faced significant challenges in malaria prevention and control.

European authors have observed poor adherence to malaria chemoprophylaxis, indicating the need for more effective preventive programs. Given the status of malaria as one of the deadliest infectious diseases, physicians should suspect malaria in every patient with fever and a history of travel to endemic areas. Due to potential lethal outcomes, severe malaria cases should be treated in experienced centers.

**Nomenclature**
ALT: alanine aminotransferase
ARDS: acute respiratory distress syndrome
AST: aspartate transaminase
CLOCC: cytotoxic lesions of the corpus callosum
COVID‐19: coronavirus disease‐19
ECMO: extracorporeal membrane oxygenation
ICU: intensive care unit
IMC: intermediate care unit
LDH: lactate dehydrogenase
P: Plasmodium
PCR: polymerase chain reaction
NO: nitric oxide
SARS‐CoV‐2: severe acute respiratory syndrome coronavirus 2
WHO: World Health Organization

## Author Contributions

P.M. and V.P. contributed to conceptualization, methodology, and analysis and wrote the original draft. P.M., S.S., S.B., J.R., B.M.J.L., and V.P. generated the data. C.K., S.S., S.B., K.Z., J.R., B.M.J.L., T.S., and M.M. contributed to writing, reviewing, and editing.

## Funding

Open Access funding enabled and organized by Projekt DEAL.

## Disclosure

All authors read and approved the final manuscript.

## Ethics Statement

This retrospective study was approved by the Institutional Review Board of the University Regensburg (Protocol Number: 23‐3222‐104). In accordance with § 15 para. One of the Professional Code for Doctors in Bavaria for retrospective research projects, the need for informed consent was waived by the Institutional Review Board of the University Regensburg. The patient data were anonymized. The study was conducted in accordance with the Declaration of Helsinki.

## Consent

The authors have nothing to report.

## Conflicts of Interest

The authors declare no conflicts of interest.

## Supporting Information

Additional supporting information can be found online in the Supporting Information section.

## Supporting information


**Supporting Information 1** Figure S1: Malaria disease around the world.

**Supporting Information 2** Figure S2: Epidemiology of imported malaria cases in Europe in 2021.

**Supporting Information 3** Figure S3: Imported malaria in Germany. Within Germany, from 08/21 until 08/22, the state of North Rhine‐Westphalia reported the most imported malaria cases. The state of Bavaria reported 95 cases.

**Supporting Information 4** Figure S4: Imported malaria in Bavaria, Germany. In Bavaria, from 08/21 until 08/22, the district of Upper Bavaria reported most malaria cases, followed by Lower Bavaria and Upper Palatinate.

**Supporting Information 5** Figure S5: Cerebral computed tomography (axial plane, native) showing profound cerebral edema in a patient with malaria tropica (a). A follow‐up CT scan after 3 days showed a markedly reduced swelling with definable gyri and sulci (b). Cerebral Magnetic resonance imaging (axial plane, T2/FLAIR sequence) showing diffuse cortical hyperintensities and swelling (c). A follow‐up MRI revealed regression of the hyperintensity of the cortex and basal ganglia after 1 week (d).

**Supporting Information 6** Figure S6: Cerebral magnetic resonance imaging (axial FLAIR weighted imaging) showing diffuse cortical hyperintensities and swelling, comparable with an edema (a). A follow‐up MRI after 5 days revealed regressive findings (b). Cerebral magnetic resonance imaging (axial diffusion weighted imaging—DWI) showing a well‐circumscribed oval lesion within the splenium, compatible with a cytotoxic lesion of the corpus callosum (CLOCC) in a patient with malaria tropica (c). A follow‐up MRI after 5 days revealed a complete regression of the finding (d).

## Data Availability

The datasets generated and/or analyzed during the current study are not publicly available due to data privacy but are available from the corresponding author on request.
