# Supplementary figures and images for: Malaria: 11‐Year Experience With Imported Cases at a German University Hospital and Epidemiological Trends Amid the COVID‐19 Pandemic
Source: J Parasitol Res. 2026 Apr 8;2026:9333826. doi: 10.1155/japr/9333826 (PMC13058724; doi:10.1155/japr/9333826)

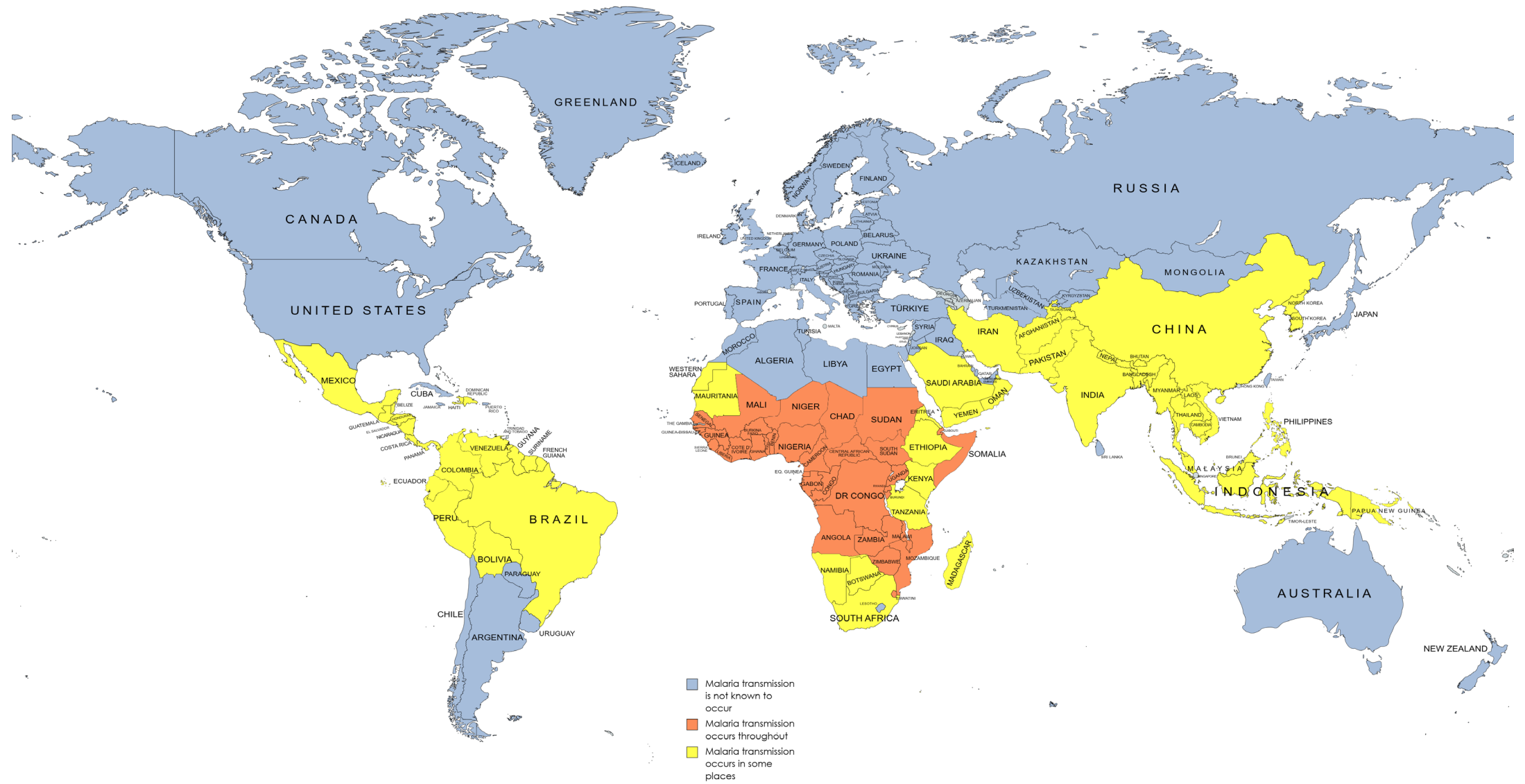

Supplementary figure 1

Supplement: Supplementary file 1 — Supporting Information 1 Figure S1: Malaria disease around the world. [file JAPR-2026-9333826-s003.pdf]

Supplementary figure 2

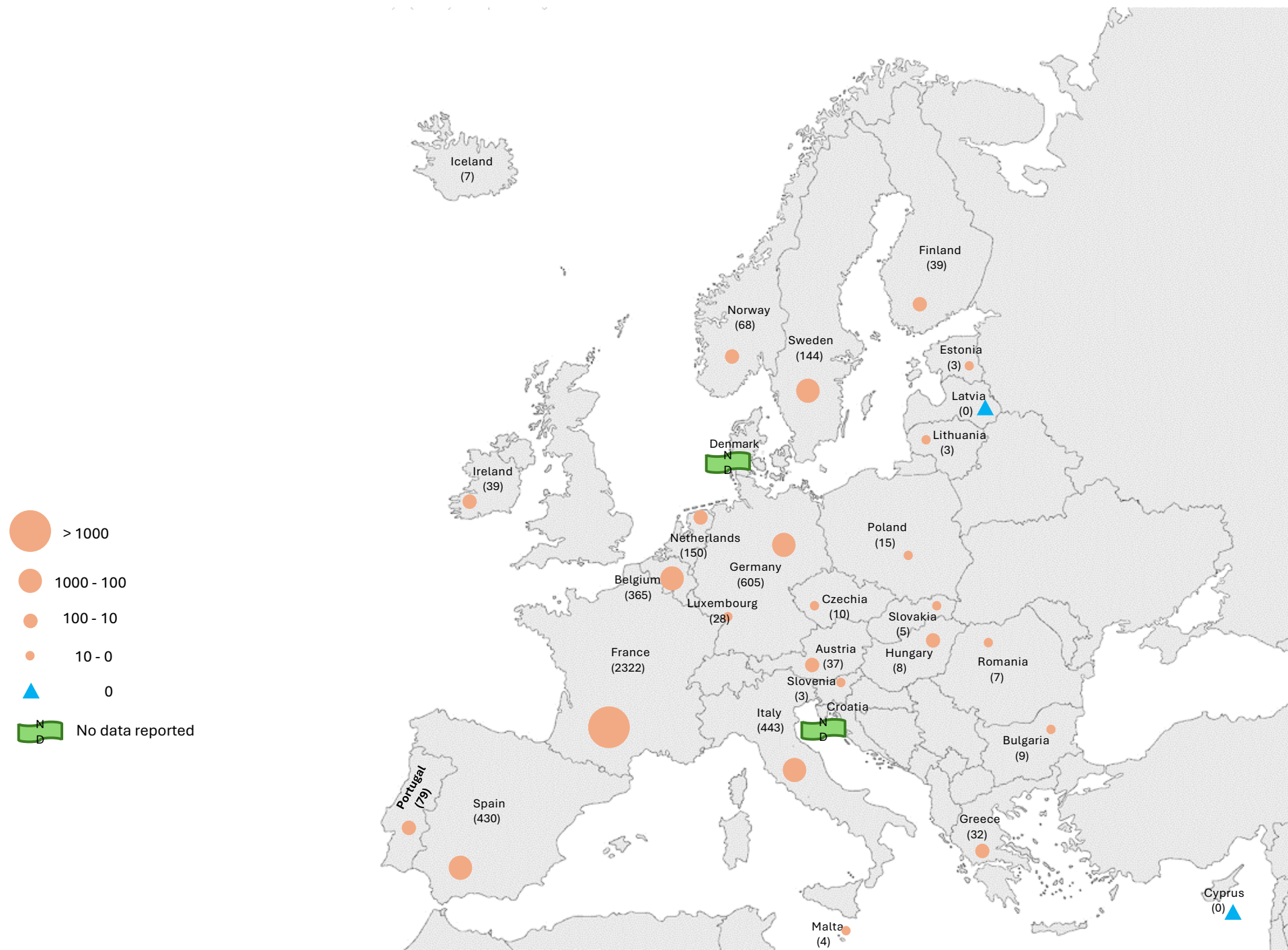

Supplement: Supplementary file 2 — Supporting Information 2 Figure S2: Epidemiology of imported malaria cases in Europe in 2021. [file JAPR-2026-9333826-s002.pdf]

Supplementary figure 3

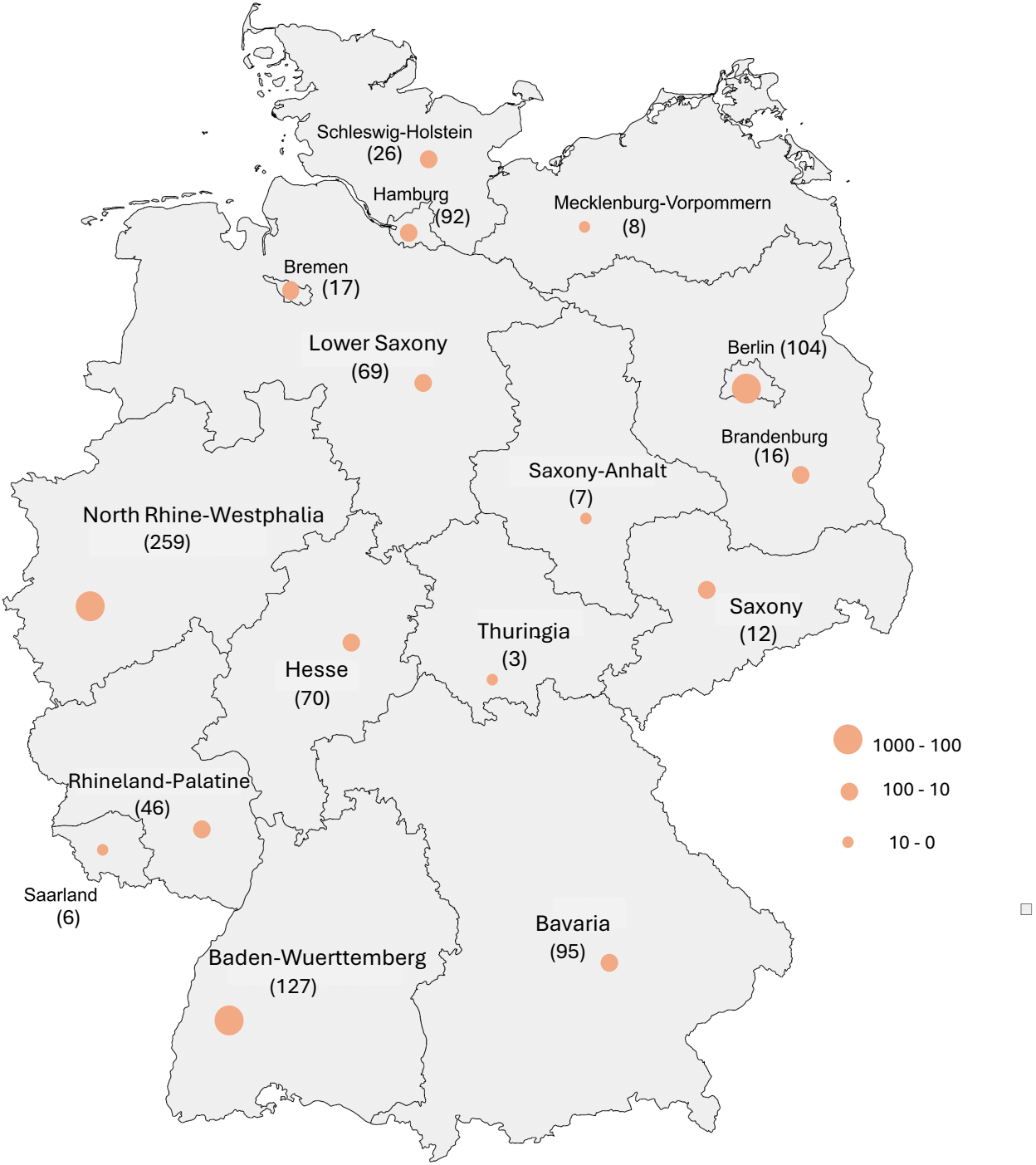

Supplement: Supplementary file 3 — Supporting Information 3 Figure S3: Imported malaria in Germany. Within Germany, from 08/21 until 08/22, the state of North Rhine‐Westphalia reported the most imported malaria cases. The state of Bavaria reported 95 cases. [file JAPR-2026-9333826-s001.pdf]

Supplementary figure 4

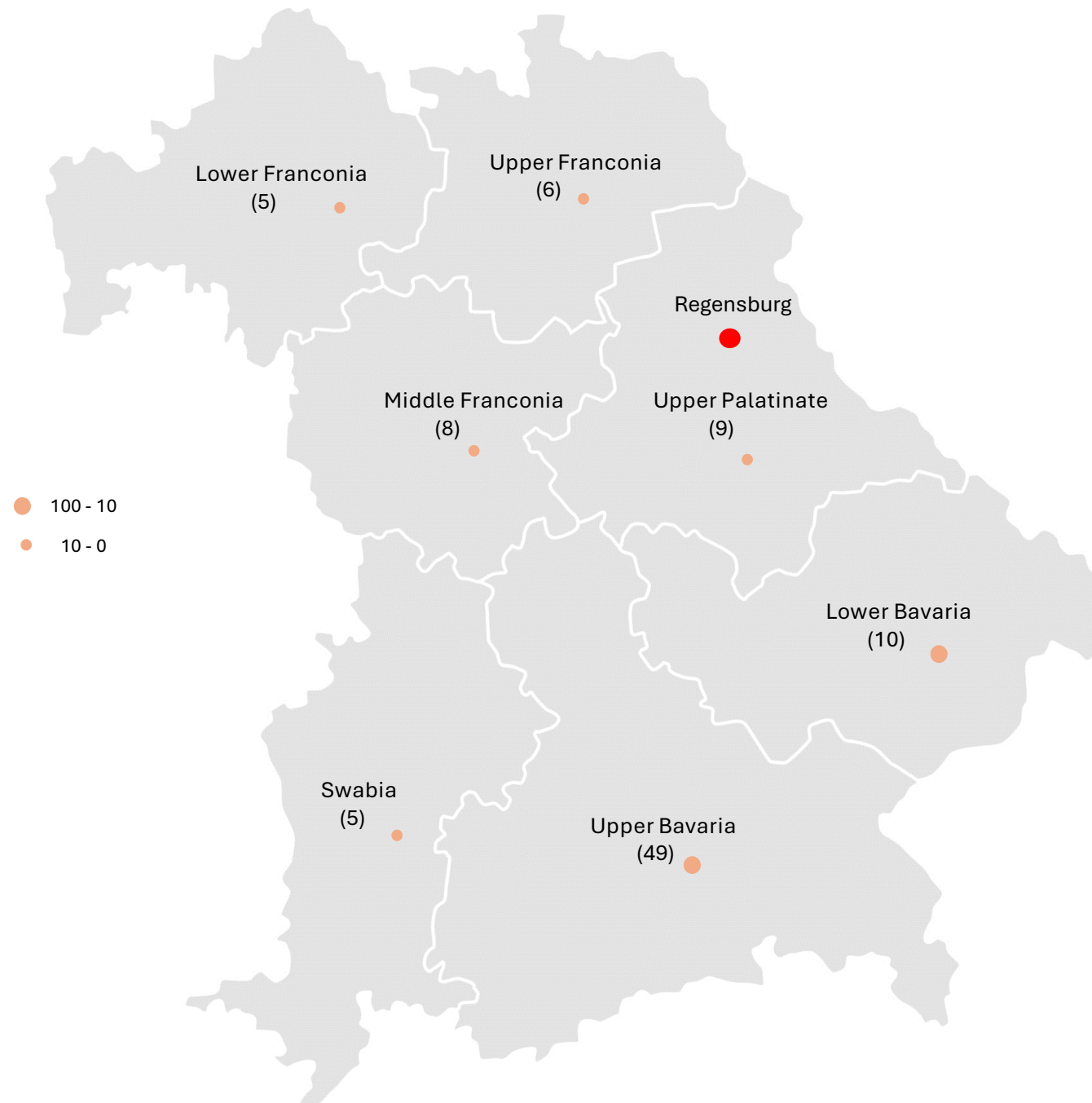

Supplement: Supplementary file 4 — Supporting Information 4 Figure S4: Imported malaria in Bavaria, Germany. In Bavaria, from 08/21 until 08/22, the district of Upper Bavaria reported most malaria cases, followed by Lower Bavaria and Upper Palatinate. [file JAPR-2026-9333826-s006.pdf]

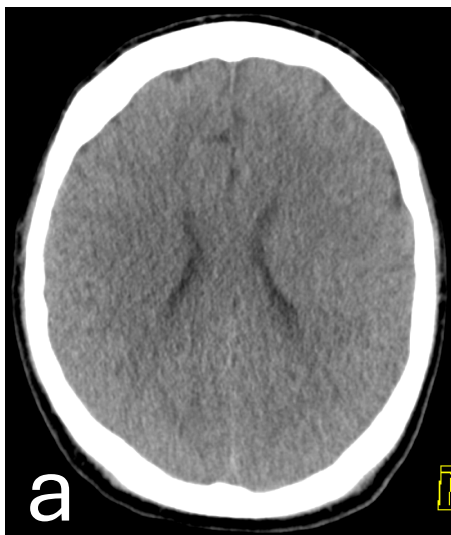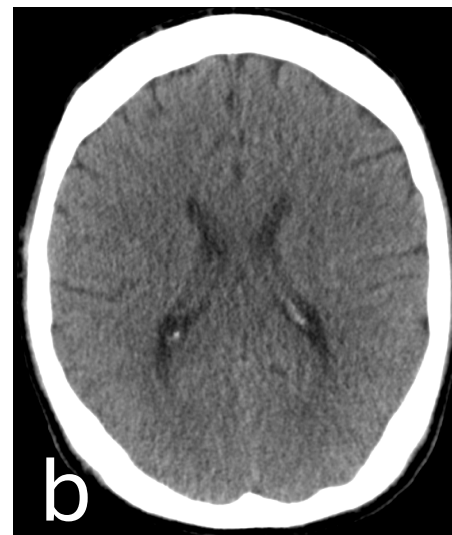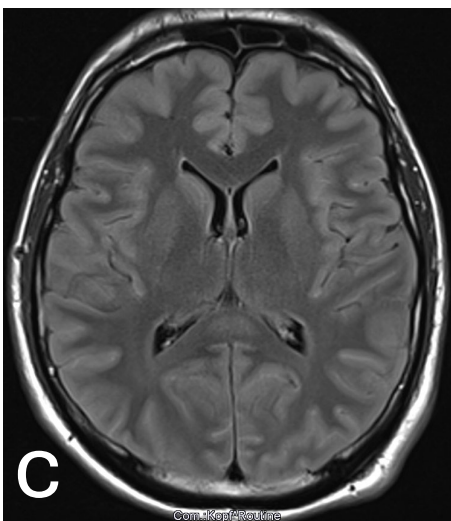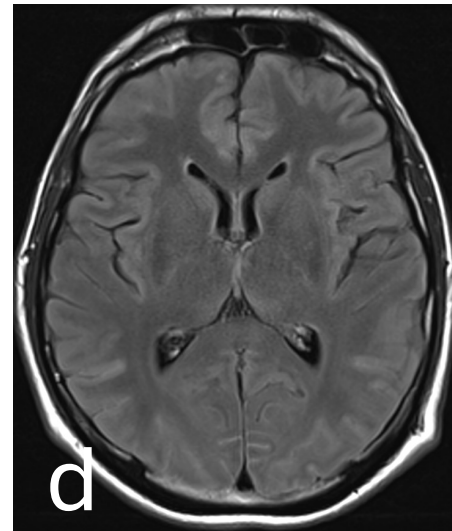

Supplementary figure 5

Supplement: Supplementary file 5 — Supporting Information 5 Figure S5: Cerebral computed tomography (axial plane, native) showing profound cerebral edema in a patient with malaria tropica (a). A follow‐up CT scan after 3 days showed a markedly reduced swelling with definable gyri and sulci (b). Cerebral Magnetic resonance imaging (axial plane, T2/FLAIR sequence) showing diffuse cortical hyperintensities and swelling (c). A follow‐up MRI revealed regression of the hyperintensity of the cortex and basal ganglia after 1 week (d). [file JAPR-2026-9333826-s005.pdf]

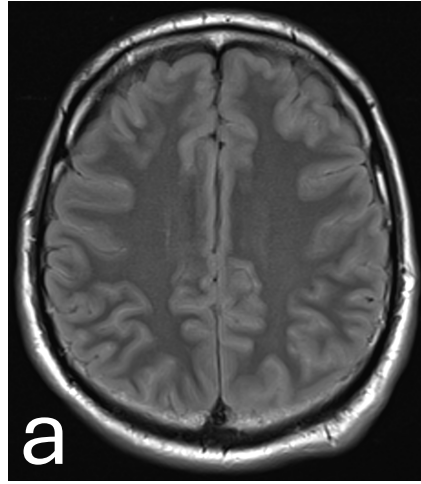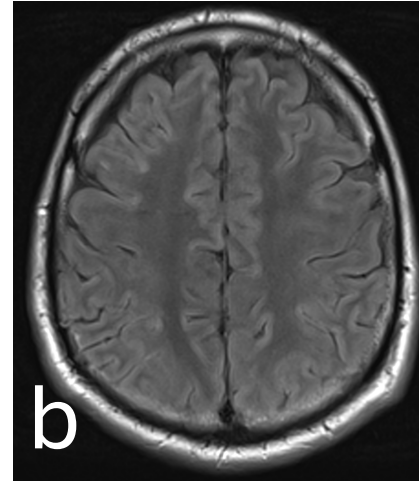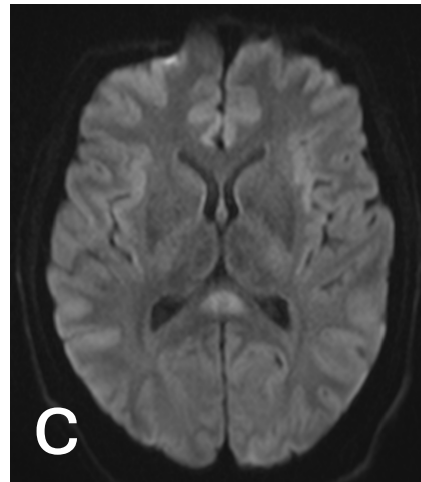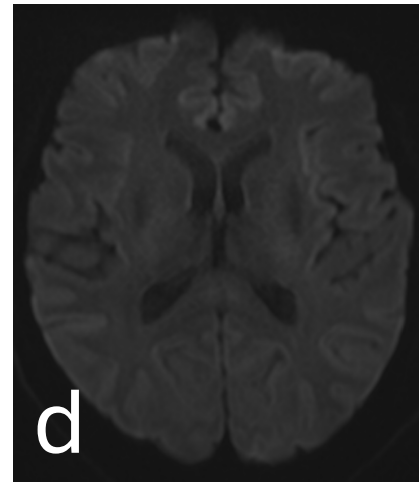

Supplementary figure 6

Supplement: Supplementary file 6 — Supporting Information 6 Figure S6: Cerebral magnetic resonance imaging (axial FLAIR weighted imaging) showing diffuse cortical hyperintensities and swelling, comparable with an edema (a). A follow‐up MRI after 5 days revealed regressive findings (b). Cerebral magnetic resonance imaging (axial diffusion weighted imaging—DWI) showing a well‐circumscribed oval lesion within the splenium, compatible with a cytotoxic lesion of the corpus callosum (CLOCC) in a patient with malaria tropica (c). A follow‐up MRI after 5 days revealed a complete regression of the finding (d). [file JAPR-2026-9333826-s004.pdf]
